# Prebiotics, Probiotics, and Synbiotics—A Research Hotspot for Pediatric Obesity

**DOI:** 10.3390/microorganisms11112651

**Published:** 2023-10-28

**Authors:** Reka Borka Balas, Lorena Elena Meliț, Ancuța Lupu, Vasile Valeriu Lupu, Cristina Oana Mărginean

**Affiliations:** 1 Department of Pediatrics I, “George Emil Palade” University of Medicine, Pharmacy, Sciences and Technology, Gheorghe Marinescu Street, No. 38, 540136 Târgu Mureș, Romania; rekaborkabalas@gmail.com (R.B.B.); marginean.oana@gmail.com (C.O.M.); 2 Department of Pediatrics, University of Medicine and Pharmacy Gr. T. Popa Iași, Universității Street No 16, 700115 Iași, Romania; anca_ign@yahoo.com (A.L.); valeriulupu@yahoo.com (V.V.L.)

**Keywords:** prebiotics, probiotics, synbiotics, child, obesity, gut microbiota

## Abstract

Childhood obesity is a major public health problem worldwide with an increasing prevalence, associated not only with metabolic syndrome, insulin resistance, hypertension, dyslipidemia, and non-alcoholic fatty liver disease (NAFLD), but also with psychosocial problems. Gut microbiota is a new factor in childhood obesity, which can modulate the blood lipopolysaccharide levels, the satiety, and fat distribution, and can ensure additional calories to the host. The aim of this review was to assess the differences and the impact of the gut microbial composition on several obesity-related complications such as metabolic syndrome, NAFLD, or insulin resistance. Early dysbiosis was proven to be associated with an increased predisposition to obesity. Depending on the predominant species, the gut microbiota might have either a positive or negative impact on the development of obesity. Prebiotics, probiotics, and synbiotics were suggested to have a positive effect on improving the gut microbiota and reducing cardio-metabolic risk factors. The results of clinical trials regarding probiotic, prebiotic, and synbiotic administration in children with metabolic syndrome, NAFLD, and insulin resistance are controversial. Some of them (*Lactobacillus rhamnosus bv-77*, *Lactobacillus salivarius*, and *Bifidobacterium animalis*) were proven to reduce the body mass index in obese children, and also improve the blood lipid content; others (*Bifidobacterium bifidum*, *Bifidobacterium longum*, *Lactobacillus acidophilus*, *Lacticaseibacillus rhamnosus*, *Enterococcus faecium*, and fructo-oligosaccharides) failed in proving any effect on lipid parameters and glucose metabolism. Further studies are necessary for understanding the mechanism of the gut microbiota in childhood obesity and for developing low-cost effective strategies for its management.

## 1. Introduction

Childhood obesity, a real pandemic, represents, in fact, a major public health problem worldwide, with an increasing trend in prevalence during the last decades, especially in developed countries [1,2,3,4,5]. Due to its associated short- and long-term complications, it carries an important burden not only for health services, but also for society, since it is associated with both psychosocial problems like bullying, resulting in school absences and consequent poor school results, and organic conditions such as metabolic syndrome with insulin resistance, cardiovascular disorders, hypertension, dyslipidemia, non-alcoholic fatty liver disease, obstructive sleep apnea, and even neoplasia [6,7,8,9,10,11,12,13,14]. According to the most recent reports of the World Health Organization (2020), 39 million children under the age of 5 years suffer from either overweight or obesity, with an impressive increase between 5 and 19 years, reaching up to 340 million cases [15]. Over the years, several studies focused on assessing the factors involved in the determinism of obesity, underlying a complex interplay between genetic background, and maternal and obesogenic factors such as maternal diet, eating habits, urbanization, or a sedentary lifestyle, but were not able to precisely state the role or the exact contribution of each of them in the complicated puzzle of obesity [16,17,18,19].

It is well-documented that, aside from bacteria, which are the main community harbored by the human intestine, the human intestine is also colonized by viruses, protozoa, and archae [20,21]. Although several studies proved that microbial DNA is present in samples of amniotic fluid, placenta, and meconium, suggesting that the colonization process might begin in utero, no unanimous consensus was reached yet in the global scientific community [22,23]. Thus, studies that assessed the role of the placental microbial composition, as well as those that compared meconium samples from different newborns, revealed important differences in terms of microbial composition, depending on maternal well-being [24,25,26,27]. Nevertheless, it is clearly established that the colonization process ends between 3–5 years of age, when the child’s microbiota resembles to that of an adult [28]. Several external factors were proven to be essential for the maturation of the microbiota composition, such as maternal diet, type of delivery and feeding, and the early use of antibiotics [6,29]. Thus, in vaginally born infants, studies revealed that facultative anaerobic bacteria, including *Escherichia* spp. and the *Enterobacteriaceae* family, along with *Lactobacillus*, are the main compounders of intestinal microbiota [30,31]. Other studies pointed out *Sneathia*, *Lactobacillus*, and *Prevotella* as the main genera colonizing the gut of term infants born vaginally [32]. Contrariwise, when born by cesarean section, the infant’s gut microbiome composition resembles that of the mother’s skin and nosocomial surroundings, consisting of lower levels of *Escherichia-Shigella*, *Bifidobacteria*, and *Bacteroidetes* when compared to vaginally born newborns, associated with an increase in *Staphylococcus*, *Corynebacterium*, and *Propionibacterium* [32,33,34,35,36,37]. The findings were correlated with an increased risk of obesity during childhood [38,39].

In addition, several differences were noticed depending on the type of feeding, consisting in a predominance of *Bifidobacteria* in breastfed infants and a more diversified microbiota in formula-fed infant with a predominance of *Bacteroides*, *Staphylococci*, *Clostridia*, *Enterobacteria*, *Enterococci*, and the genus *Atopobium* [6]. Human milk oligosaccharides were proven to favor the growth of *Bifidobacteria* and *Bacteroides*, acting also as modulators of innate immune responses, anti-inflammatory molecules, and prebiotics [40]. Moreover, maternal health is a crucial modulator of human milk composition with potential negative consequences on the infant’s gut microbiota [33]. Thus, maternal obesity was associated with a decrease in bacterial community diversity, resulting in higher levels of *Staphylococcus* and decreased levels of *Bifidobacterium* [41,42].

Important changes in microbiota composition occur following the introduction of complementary feeding, such as a considerable increase in alpha diversity due to the replacement of *Actinobacteria* and *Proteobacteria* with the phyla *Bacteroidetes* and *Firmicutes*, along with a significant increase in the production of short-chain fatty acids [43]. Thus, major changes in the gut microbial composition were noticed between 9 and 18 months of age, consisting in an increase of *Bacteroides* and *Clostridium* species, along with a decrease of *Bifidobacteria*, *Enterobacteriaceae*, and *Lactobacili* [44]. Gut microbial diversity and composition were proved to differ depending on the type of diet, with the suggestion that a high-fat Westernized diet might be related to an increased risk of developing obesity during childhood due to a decrease in *Prevotellaceae* [45]. The same study pointed out that, if consuming a carbohydrate-based diet, children experience a decrease in *Bacteroideaceae* and an increase in *Prevotellaceae* [45].

The aim of this review was to assess the differences and the impact of gut microbial composition on several obesity-related complications such as metabolic syndrome, non-alcoholic-fatty liver disease, or insulin resistance.

## 2. Obesity, Microbiota, and Prebiotics, Probiotics, and Synbiotics

It is well-known that both childhood obesity and its related metabolic complications are increasing in incidence during the last decades, but, unfortunately, the molecular basis of the relationship between body weight and its metabolic implications is not completely understood. Still, the most commonly reported obesity-associated metabolic disorder is insulin resistance, which, in fact, is the trigger of subsequent type 2 diabetes mellitus and cardiovascular diseases [4,7,8]. Therefore, the implications of pediatric obesity go beyond childhood into adulthood, increasing considerably the health-related costs. Other important long-term complications of obesity consist of chronic inflammation, which was proven to be present also in children [46], dyslipidemia, hypertension, hyperuricemia, obstructive sleep apnea, nonalcoholic fatty liver disease, and even cancer [6]. Although genetic background plays a major role in predisposing individuals to obesity, obesogenic factors such as eating habits, sedentary lifestyle, and urbanizations seem to be the ones that decide, finally, whether an individual will become obese or not [16]. We must also acknowledge that not all individuals with obesity display the same degree of metabolic-related complications, although these were proven to occur early during childhood in others. Thus, recently, several authors felt the need to distinguish between these two categories of obese individuals by defining two terms: metabolically ‘healthy’ obese (MHO) and metabolically ‘unhealthy’ obese (MUO) [47]. Moreover, it was proven that MHO might migrate into the MUO phenotype during puberty [48] (Table 1).

Aside from the external obesogenic factors, studies suggested that the microbiota itself might be obesogenic, proving that fecal transplantation from an obese donor increases the risk of weight gain in the case of germ-free recipients following the same diet [49]. The colonization of the human intestine begins during birth and continues until the age of 3–5 years when it resembles that of an adult reaching maturity [50]. Thus, in the setting of vaginal delivery, the infant’s microbiome is similar to that of the maternal vaginal microbiota consisting of *Escherichia* spp. and *Lactobacillus*, but also other members of the *Enterobacteriaceae* family [30,31]. Within the first day after birth, the infant’s gut begins to be colonized with *Bifidobacterium* and *Clostridium* [31], which, in fact, is a consequence of breastfeeding, as it is well-documented that breastfed infants have higher levels of *Bifidobacteria* compared to those fed with a formula, whose microbiota is more diversified, including Staphylococci, *Clostridia*, *Bacteroides*, *Enterobacteria*, *Enterococci*, and the genus *Atopobium* [6]. Although important changes in the microbiome composition occur after the introduction of solid feeding, it seems that the microbiota assessed at the age of three months might be considered a more reliable predictor of the future risk of increased weight gain than the profile assessed at the age of 12 months [43]. In fact, the infant’s microbiota at the age of three months is mainly composed of *Bifidobacteriaceae*, *Bacteroidaceae*, *Enterobacteriaceae*, *Lachnospiraceae*, *Veillonellaceae*, and *Ruminococcaceae* [43]. Nevertheless, once complementary feeding is introduced, an important maturation stage occurs within the infant’s microbiota, resulting in an important increase in alpha diversity, consisting of *Proteobacteria* and *Actinobacteria* replacement by the phyla *Bacteroidetes* and *Firmicutes*, as well as a considerably increased production of short-chain fatty acids (SCFAs) [43] (Table 1).

Several mechanisms were hypothesized regarding the role of intestinal microbiota in the development of obesity, such as the ability of intestinal microbes to extract energy from nondigestible polysaccharides resulting in an overproduction of SCFAs and subsequent impaired food absorption, a decrease in the AMP kinase with a decreased oxidization of fatty acids in the muscle, and a higher hepatic lipogenesis mediated via ChREBP/SREBP-1, along with an altered metabolism of bile acids affecting the effective transport of cholesterol [6]. In addition, it was also noticed that hormonal-mediated mechanisms participated in the pathogenesis of obesity via glucagon-like peptide 1 (GLP-1) and GLP-1 receptors G protein-coupled 43 and 41 (GPR41 and GPR43). In fact, GPR41 is involved in the synthesis of the intestinal anorexigenic hormone called peptide YY, responsible for regulating satiety, decreasing gastric emptying, and reducing intestinal transit time, as well as for increasing energy harvest, along with hepatic lipogenesis, while GPR43 has the ability to sense postprandial energy excess and to regulate energy expenditure [51,52,53]. A less studied relationship between the host energy metabolism is mediated by fasting-induced adipocyte factor (FIAF), which, if inhibited by gut microbes, leads to no inhibitory action on lipoprotein lipase (LPL) and the subsequent accumulation of adipocyte within peripheral tissues [54].

In terms of the bacterial composition of the gut microbiota, several studies indicated that, depending on the predominant species, it might have either a positive or negative impact on the development of obesity. Therefore, among the bacteria that were associated with a lower body mass index, studies pointed out *Fecalibacterium prausnitzii* and *Clostridium difficile*, *Staphylococcus genus*, or *Bacteroidetes phylum* [55]. Nevertheless, in term of *Bacteroidetes*, the findings reported in the current studies are rather controversial. Thus, a systematic review indicated a possible positive association between obesity and *B. fragilis*. Contrariwise, in the same review, the authors pointed out that other bacteria belonging to the *Bacteoridetes phylum*, i.e., *Bacteroidetes* and *Prevotella*, were less abundant in obese subjects in spite of their role as triggers of intestinal inflammation [55]. When assessing the entire *Bacteroidetes phylum*, the authors proved a decrease of their levels in children with obesity in comparison to the control group [55]. Similar to *B. fragilis*, *C. leptum* and *E. hallii* were also proven to be more abundant in obese/overweight infants and preschool/school-aged children [55]. All these findings reported in the previously mentioned studies were all related to the role of these bacteria as promoters or suppressors of inflammation [55] (Table 1).

Early disruptions in microbial gut compositions, triggered especially by the administration of antibiotics, were proven to be associated with an increased predisposition to obesity [56]. An animal model study proved that the exposure of mice to low-dose penicillin induces important changes in the gut microbiota, which, if transplanted to germ-free recipients, trigger body fat deposition, weight gain, and the severe impairment of gut immune system functionality, consisting in diminishing the ileal expression of genes responsible for encoding antimicrobial peptides, as well as those which regulate T helper 17 cell populations [57]. Moreover, repeated administrations of antibiotics have the same effect in terms of weight gain, increasing the bone mineral content and growth as well due to microbiome perturbations, which, in fact, are directly related to the classes of antibiotics and the number of pulses [58]. Aside from antibiotics, a study performed on children from the United States also revealed the same effect on weight gain in the setting of proton pump or histamine type 2 receptor antagonists administered within the first two years of life, the association being stronger if multiple classes of antibiotics are prescribed, or if the course of therapy is prolonged [59].

Nutritional interventions including prebiotics, probiotics, and synbiotics were suggested to have a positive effect on improving the gut microbiota, reducing cardio-metabolic risk factors, such as obesity [60,61]. Thus, the modulation of intestinal microbiome composition using these compounds was recently suggested to have a major positive impact on obesity-associated gut dysbiosis [62]. Prebiotics represent non-digestible substrates meant to favor the development of beneficial bacterial species, which are selectively utilized by the host’s micro-organisms [63]. Probiotics contain live micro-organisms carefully selected in adequate amounts [64], while synbiotics represent a mixture of pre- and probiotics [65]. Thus, based on the aforementioned crosstalk between obesity and gut microbiota, studies tried to assess the impact of different bacteria supplementation on body weight. *Akkermansia muciniphila* is one of the bacteria that proved to be effective in both animal and human model studies. In a study performed on mice, *Akkermansia muciniphila* administration reversed insulin resistance and weight gain as a result of a high-fat diet [66], most likely due to its outer membrane protein which interacts with toll-like receptor 2 in order to improve the alterations of the gut barrier, resulting in the normalization of body weight and insulin resistance [67]. Moreover, a randomized, placebo-controlled, double-blind study involving subjects with overweight/obesity and insulin resistance pointed out that a supplementation of 10^10^ *Akkermansia muciniphila* per day for three months was associated with a decrease in plasma insulin and cholesterol levels, improving insulin sensitivity [68]. In spite of the lack of significance, the study also underlined that this bacterium might also have a positive effect on fat mass, hip circumference, and body weight. Similar results were also reported for *Christensenella minuta* in a study performed on mice [69]. The supplementation of *Lactobacillus rhamnosus strain GG* and *Lactobacillus salivarius* were also assessed in children or adolescents with overweight and obesity, but, although the first lead to important changes in gut microbial composition, none of them displayed an effect on the body mass index z score, or body fat mass [70,71,72]. Despite their lack of effectiveness when administered separately, a recent study also involving pediatric subjects pointed out that the supplementation of multi-strain probiotics consisting of *Lactobacillus salivarius* AP-32, *Lactobacillus rhamnosus bv-77*, and *Bifidobacterium animalis CP-9* resulted in a lowered serum total cholesterol, low density lipoprotein, tumor necrosis factor-alpha, and leptin levels, and a reduced body mass index, but, at the same time, induced an elevation of adiponectin and high-density lipoprotein [73]. Moreover, the authors suggested that the positive effects on the lipid pathway might be related to *Lactobacillus* spp., while those on adiponectin might be related to *Bifidobacterium animalis*. Verma et al. also sustained that compounds that contain multiple species of probiotics (*Lactobacillus plantarum* DSM 24730, *Lactobacillus plantarum* DSM 24731, *Lactobacillus plantarum* DSM 24735, *Lactobacillus plantarum* DSM 24801, *Lactobacillus paracasei* DSM 24737, *Lactobacillus salivarius* DSM 24800, *Lactobacillus delbrueckii* DSM 25998, *Bifidobacterium animalis* DSM 24736, *Bifidobacterium breve* DSM 24732, and *Pediococcus pentosaceus* DSM 24734) might be effective in improving the gut microbial composition and fasting glucose levels in adolescents with severe obesity [74].

However, probiotics might not work as preventive therapies for obesity, according to a study in which the authors administered *Lactobacillus paracasei F19* to 120 infants from the age of 4 to 13 months, during weaning, and after a close follow-up of this population, and noticed no modulatory effect of this probiotic on either growth or body composition at school age [75]. The findings reported in studies using *Lactobacillus* spp. as probiotics might differ even between pediatric and adult populations, according to a meta-analysis which observed that adults that were administered this probiotic experienced weight loss, while children presented minor weight gain [76]. Nevertheless, Luoto et al. defined two phases in the development of obesity—an initial one beginning in the perinatal phase to 48 months of age, and a second one after the age of 4 years—and stated that, if *Lactobacillus rhamnosus GG* is supplemented in future mothers 4 weeks before delivery is expected and in infants until the age of 6 months, the children will have a healthy growth pattern, but the effect lasts only during the first phase of obesity, with no effect at 10 years of age [77] (Table 1).

It is well-documented that children born to mothers with overweight and obesity have a higher risk of becoming obese later in life [50,78,79,80]. Therefore, this risk might be lowered with proper nutritional intervention even during pregnancy. According to Saros et al., probiotics (*Lactobacillus rhamnosus* HN001 and *Bifidobacterium animalis* ssp. *lactis 420*) administration alone or combined with fish oil during pregnancy and in the first 6 months postpartum decreased the risk of obesity in their offspring at the age of 24 months [81]. Interestingly, recent evidence suggests that probiotics administered in pregnant women might act as a gene-targeted therapy since they might significantly decrease the DNA methylation in certain gene promoters of obesity in both mothers and their children, subsequently decreasing the risk for excessive weight gain [82]. Moreover, breast-milk-derived probiotics such as *Lactobacillus plantarum 73a* and *Bifidobacterium animalis* subsp. *lactis INL1* were also suggested to act as potential candidates in the management of pediatric obesity according to the study of Oddi et al., who proved that the administration of these two probiotics isolated from breastmilk results in a decrease of *phylum Proteobacteria* and *genera Shigella*, *Escherichia*, and *Clostridium sensu stricto 1*, which are all known to trigger obesity and its associated inflammatory status or insulin resistance [83].

In terms of prebiotics, a clinical trial designed for improving the microbiota in obese individuals, including 48 healthy overweight and obese adults, assessed the administration of 21 g of oligofructose compared to a placebo every day for 12 weeks, and concluded that oligofructose favors weight loss, reduces calorie intake and the levels of ghrelin, also known as the hunger hormone, improves glucose regulation, and increases the concentrations of satiety hormone YY [51]. Another clinical trial including 42 children with overweight and obesity using oligofructose-enriched inulin administered daily for 16 weeks in a dose of 8 g/day indicated that it might be associated with a reduced weight gain, and improved truncal fat disposition and body weight z-scores, as well as lower levels of interleukin-6 and triglycerides, when compared to a group receiving an isocaloric placebo [52]. In addition, the authors also noticed an increase of fecal *Bifidobacterium* spp. in the overweight/obese group after the administration of oligofructose-enriched inulin. Other microbiota-targeting therapies for obesity involving prebiotics used fibers as supplements and noticed that individuals receiving high-dietary-fiber supplements for 12 weeks expressed an increased abundance of SCFA-producing bacteria within their gut microbial composition, associated also with improved fasting and postprandial blood glucose levels, as well as hemoglobin A_1c_ [53] (Table 1).

**Figure 1:**
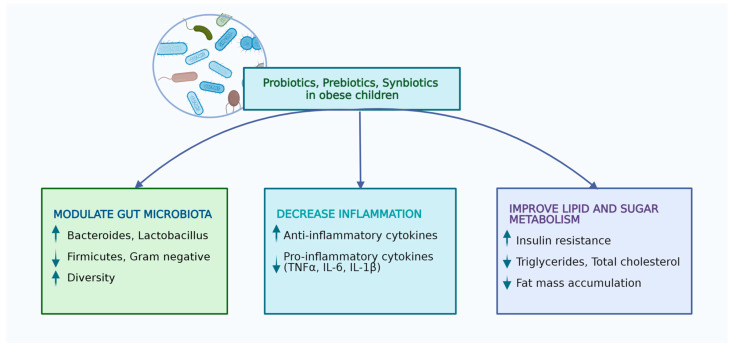
Effects of probiotics, prebiotics, and synbiotics in obese children (performed with Biorender licence).

Synbiotics represent compounds that have the same properties of prebiotics and probiotics, together promoting probiotic survival in the gastrointestinal tract, and they might result in a better outcome regarding the host’s health when compared to prebiotics and probiotics separately [84,85]. A recent study performed on 60 children with overweight and obesity aged between 8 and 18 years assessed the combination between *Lactobacillus indicus* and *Lactobacillus coagulans* as probiotics, combined with short-chain fructo-oligosaccharide as a prebiotic, and noticed a significant reduction of waist–height ratio in children receiving this combination compared to those receiving the placebo [86]. Nevertheless, another study involving obese children and teenagers pointed out a decrease in weight and body mass index after one month of supplementation with synbiotics [87]. Similarly, a meta-analysis of nine randomized controlled trials proved that synbiotic supplementation in children and adolescents with obesity might be associated with a significant decrease in the body mass index z score [88]. Kilic Yildirim et al. conducted a randomized, double-blind, placebo-controlled trial on 61 children with exogenous obesity and pointed out that the group which received a synbiotic supplement once daily, consisting of a mixture of probiotics, including *Lacticaseibacillus rhamnosus*, *Lactobacillus acidophilus*, *Bifidobacterium longum*, *Bifidobacterium bifidum*, and *Enterococcus faecium*, combined with fructo-oligosaccharides, for 12 weeks presented significantly greater changes in weight, body mass index, and anthropometric parameters like waist circumference and waist-circumference-to-height ratio when compared to the placebo group [89]. Thus, the authors concluded that a multispecies synbiotic intake, along with exercise and diet, might represent an effective weight-loss strategy for pediatric obesity. Similar studies performed on adults revealed contradictory effects of synbiotics on both the body mass index, and body fat or anthropometric parameters in obese subjects [90,91] (Table 1). The impact and mechanism through which probiotics improve the wellbeing of children with obesity are related in Figure 1.

**Table 1:** The effects of prebiotics, probiotics, and symbiotic on children’s obesity.

Reference (Author, Year)	Type of Study	Study Population	Objectives/Outcomes Measured	Prebiotics, Probiotics, and Synbiotic	Conclusions/Outcome
Vajro et al., 2011 [70]	Double-blind, placebo-controlled pilot study	*n* = 20 (10 probiotic/10 placebo) Age: 9–13 years BMI > 95th percentile	Effects of short-term probiotic on children with obesity-related liver disease	*Lactobacillus rhamnosus GG* (12 billion CFU/day) for 8 weeks	Decrease in alanine aminotransferase and in antipeptidoglycan polysaccharide antibodies, irrespective of changes in BMI z score and visceral fat.
Gobel et al., 2012 [71]	Double-blind placebo-controlled trial	*n* = 50 (27 *LS-33*/23 placebo) Age: 12–15 years BMI > 95th percentile	Effect of *Lactobacillus salivarius Ls-33* on biomarkers related to inflammation and metabolic syndrome (MS)	*Lactobacillus salivarius Ls-33* (10^10^ CFU/day) for 12 weeks	No effects of probiotic strain *Ls-33* on either the inflammatory markers or the markers of MS.
Marcelo et al., 2022 [72]	Non-randomized controlled, prospective, double-blind interventional clinical trial	*n* = 44 (22 probiotic/22 placebo) Age: 8–17 years BMI > 95th percentile	Impact of probiotic supplementation therapy on anthropometric values and body composition	*Lactobacillus rhamnosus IAL 1883* for 6 months	Supplementation with *Lactobacillus rhamnosus IAL 1883* was not effective for weight loss or improving the body composition.
Chen et al., 2022 [73]	Double-blind, randomized, placebo-controlled trial	*n* = 53 (27 probiotic/26 placebo) Age: 6–18 years BMI ≥ 85th percentile	Effects of multi-strain probiotics on the gut microbiota and weight control/BMI, LDLC, HDLC, adiponectin, leptin	Multi-strain probiotics consisting of *Lactobacillus salivarius* AP-32, *Lactobacillus rhamnosus bv-77*, and *Bifidobacterium animalis CP-9* for 12 weeks	Lowered serum TC, LDLC, TNF-α, and leptin, and reduced BMI. Elevation of adiponectin and HDLC.
Verma et al., 2021 [74]	Randomized, double-blind, placebo-controlled pilot study	*n* = 15 (8 probiotic/7 placebo) Age: ≥13 years BMI ≥ 99th percentile	Effect of probiotics on gut microbiota and insulin resistance	Visbiome^®^ containing probiotics (*Lactobacillus plantarum* DSM 24730, *Lactobacillus plantarum* DSM 24731, *Lactobacillus plantarum* DSM 24735, *Lactobacillus plantarum* DSM 24801, *Lactobacillus paracasei* DSM 24737, *Lactobacillus salivarius* DSM 24800, *Lactobacillus delbrueckii* DSM 25998, *Bifidobacterium animalis* DSM 24736, *Bifidobacterium breve* DSM 24732, and *Pediococcus pentosaceus* DSM 24734) two sachets/day 12 weeks	Improve fasting glucose levels and gut microbial composition (decrease F/B ratio).
Karlsson et al., 2015 [75]	Double-blind, randomized, placebo-controlled intervention trial	*n* = 120 (58 probiotic/62 placebo) Age: 8–9 years	Long-term effect of feeding with *Lactobacillus paracasei F19 (LF19)* on body composition, growth, and metabolic markers (TC, HDLC, apo A-1, apo B, TG, glucose, AST, ALT, S-insuline)	Administration of *Lactobacillus paracasei F19* from 4 to 13 months of age, 1 × 10^8^ CFU/at least once daily	LF19 had no modulatory effect on growth (BMI z-score, sagittal abdominal diameter, fat-free mass, fat mass index, truncal fat %, android or gynoid fat %) or body composition at school age and no long-term impact on metabolic markers.
Luoto et al., 2010 [77]	Randomized, double-blind, prospective follow-up study	*n* = 113 (77 probiotics/82 placebo) Age: 2 weeks–10 years	Impact of perinatal probiotic intervention on childhood growth patterns and the development of overweight during a 10-year follow-up	Administration of *Lactobacillus rhamnosus* (1 × 10^10^ CFU) in mothers 4 weeks before delivery and in infants until the age of 6 months	Early probiotics may restrain excessive weight gain during the first years of life.
Saros et al., 2023 [81]	Double-blind, placebo-controlled randomized trial	*n* = 439 pregnant women BMI ≥ 25 kg/m^2^ pre-pregnancy *n* = 330 children Age ≤ 2 years 4 parallel groups: fish oil + placebo, probiotics + placebo, probiotics + fish oil, placebo + placebo	Fish oil and/or probiotic effect in pregnant women with overweight or obesity on the tendency of their 24-month-old children to become overweight	Administration of *Lactobacillus rhamnosus* HN001 and *Bifidobacterium animalis* ssp. *lactis 420* (10^10^ CFU/day) alone or combined with fish oil during pregnancy and in the first 6 months postpartum	Decreased the risk of obesity in their children at the age of 24 months.
Nicolucci et al., 2017 [52]	Randomized, double-blind, placebo-controlled trial	*n* = 42 (22 OI/20 placebo) Age: 7–12 years BMI ≥ 85th percentile	Effects of prebiotics on body composition, markers of inflammation, bile acids in fecal samples, and composition of the intestinal microbiota	Administration of oligofructose-enriched inulin (OI) 8 g/day for 16 weeks	OI administration reduces body weight z-score, percent body fat, percent trunk fat, serum interleukin 6, and triglycerides; increase fecal *Bifidobacterium* spp.
Atazadegan et al., 2023 [86]	Randomized double-blind, placebo-controlled trial	*n* = 60 (30 synbiotic/30 control) Age: 8–18 years BMI ≥ 85th percentile	Effect of synbiotic on anthropometric indices and body composition	Combination of *Lactobacillus indicus* 6 × 10^9^ colony forming units (CFU) and *Lactobacillus coagulans* 6 × 10^9^ CFU twice a day as probiotics combined with short-chain fructo-oligosaccharide for 8 weeks	A significant reduction of waist–height ratio.
Ipar et al., 2015 [87]	Open-label, randomised, controlled study	*n* = 86 (43 synbiotic/43 control) Age: 5–17 years BMI > 95th percentile	Effects of a synbiotic on anthropometric measurements, lipid profile, and oxidative stress parameters	A probiotic mixture including *Lactobacillus acidophilus*, *Lactobacillus rhamnosus*, *Bifidobacterium bifidum*, *Bifidobacterium longum*, and *Enterococcus faecium*, combined with fructo-oligosaccharydes for one month	Decrease of weight and BMI. Decrease of serum total cholesterol, low density lipoprotein cholesterol and total oxidative stress levels.
Kilic Yildirim et al., 2022 [89]	Randomized, double-blind, placebo-controlled trial	*n* = 61 (30 synbiotic/31 placebo) Age: 8–17 years BMI > 95th percentile	Effects of synbiotic on anthropometric measurements, glucose metabolism, and lipid parameters	Synbiotic including *Lacticaseibacillus rhamnosus*, *Lactobacillus acidophilus*, *Bifidobacterium longum*, *Bifidobacterium bifidum*, and *Enterococcus faecium*, combined with fructo-oligosaccharides for 12 weeks vs. placebo.	Decreased weight, BMI, and anthropometric parameters.

Legend: ALT: alanine transaminase; apo A-1: apolipoprotein A-1; apo B: apolipoprotein B; AST: aspartate transaminase; BMI: body mass index; CFU: colony forming units; F/B ratio: firmicutes-to-bacteroidetes; HDLC: high-density lipoprotein cholesterol; LDLC: low-density lipoprotein cholesterol; SHIME^®^: Simulator of the Human Intestinal Microbial Ecosystem; TC: total cholesterol; TG: triglycerides; TNF-α: tumor necrosis factor-alpha.

## 3. Implications in Obesity-Related Complications

### 3.1. Non-Alcoholic Fatty Liver Disease (NAFLD) and Non-Alcoholic Steatohepatitis (NASH)

The average prevalence of NAFLD in the general pediatric population is 7.6%, but, when assessing only the subgroup of obese children, it reaches up to 34.2%, affecting more commonly the male gender [92]. NAFLD comprises a wide spectrum of hepatic disorders, varying from isolated steatosis and liver fibrosis to cirrhosis, resulting eventually in the development of hepatocellular carcinoma. This condition is recognized when more than 5% of hepatocytes develop steatosis, noticed either radiologically or histologically without the presence of other causes that might trigger it, such as hereditary liver disorders, viral hepatitis, or increased alcohol consumption [93]. NASH is a more severe form of liver impairment consisting of hepatocyte degeneration, presenting a balloon shape associated with diffuse inflammatory infiltrate within the hepatic lobules plus/minus fibrosis [94]. Taking into account the fact that the liver is primarily exposed to the toxic products of the gut such as damaged metabolites, bacteria, and their products [95], it is not surprising that several studies tried to find the relationship between the gut microbial composition and the occurrence of NAFLD. Thus, certain authors noticed that the gut microbiota signature is different for each stage of NAFLD [96], as proven by the fact that species like *Escherichia*, *Enterobacteria*, *Proteobacteria*, and *Bacteroides* are more commonly found in patients with NASH when compared to matched controls [97]. The evidence regarding the role of probiotics, prebiotics, and synbiotics in children with NAFLD is limited. Nevertheless, Vajro et al. assessed the role of *Lactobacillus rhamnosus* in 20 children with obesity, increased alanine-aminotransferase (ALT), and the suspicion of hepatic steatosis, assessed by ultrasound, and noticed a significant decrease of ALT in children who were supplemented with this probiotic [70]. Another study, which included a larger sample of 48 children with NAFLD diagnosed by liver biopsy, concluded that multispecies-based probiotics including *Streptococcus thermophilus*, *lactobacilli*, and *bifidobacteria* might improve the ultrasound of the liver after 4 months of administration [98]. Similar findings were reported by Famouri et al., who used a combination of *Lactobacillus rhamnosus*, *Lactobacillus acidophilus*, *Bifidobacterium lactis*, and *Bifidobacterium bifidum* for 12 weeks and concluded that obese children treated with this probiotic presented significantly lower levels of ALT, as well as an amelioration of liver steatosis if compared to the placebo group [99]. Nevertheless, the randomized clinical trial of Rodrigo et al. failed in identifying any benefits of probiotics over lifestyle regarding the improvements of either laboratory parameters such as fasting blood glucose, liver function, lipid profile, and C-reactive protein, or ultrasound ones [100]. The study included 84 children with obesity, an aspartate aminotransferase/alanine aminotransferase ratio below 1, and hepatic steatosis between the I and III stages, who were randomized into two groups: the probiotic group, who were administered a probiotic with 14 bacterial strains associated with diet and physical activity, and the placebo group, who had the same regimen in terms of diet and physical activity, but received a placebo capsule. Nevertheless, the authors underlined a significant decrease of body mass index irrespective of the group, concluding that they could not find any added effect of probiotics on this finding [100]. The role of synbiotics (*Bifidobacterium longum*, *Bifidobacterium bifidum*, *Enterococcus faecium*, *Lactobacillus acidophilus*, *Lacticaseibacillus rhamnosus*, and fructo-oligosaccharides) were assessed by Yildirim and coworkers, who found no impact of this compound on NAFLD associated to obesity in children [89] (Table 2).

### 3.2. Metabolic Syndrome and Insulin Resistance

Recently, NAFLD was described as a manifestation of metabolic syndrome [101,102]. The prevalence of metabolic syndrome in the pediatric population varies between 1.2% and 22.6%, increasing to up to 60% in children with overweight and obesity [103]. Moreover, the prevalence was reported to be higher in boys and increases with age [104]. Recent evidence from the review of Fiore G. et al. sustains the beneficial role of pre-, pro-, and synbiotics in children with obesity and metabolic disorders [105]. The role of probiotics in the management of childhood metabolic syndrome was assessed in studies performed on both mothers and their infants, suggesting that the benefits of these supplements consist of regulating maternal obesity and increased blood glucose levels, as well as modulating the infant’s gut microbial composition through the placenta or breastmilk [106,107,108,109]. Unfortunately, two randomized clinical trials that assessed the impact of *Lactobacillus rhamnosus GG* and *Lactobacillus paracasei* ssp. *F19* on at least one of the components of metabolic syndrome in children aged between 2 and 19 years failed to identify significant differences between the probiotic and the placebo group [75,77]. Recent evidence highlighted the role of *Blautia* species in modulating glucose metabolism in children [110]. Although certain species were associated with a negative effect due to their proinflammatory responses such as *Blautia cocoides*, others were, on the contrary, proven to exert anti-inflammatory effects including *Blautia wexlerae* and *Blautia luti* [110,111]. Taking into account the reduction of the latter two species in obese children, studies proved that dietary interventions including arabinoxylans added to wheat bran extract resulted in an increase of these bacterial species in the bacterial community of these pediatric patients [112,113]. Another larger interventional study included 101 obese participants with insulin resistance aged between 6–18 years and concluded that *Bifidobacterium breve B632* and *Bifidobacterium breve BR03* administered for 8 weeks leads to a significant improvement in insulin sensitivity, being also associated with lower levels of fasting insulin and alanine aminotransferase, but also decreased body mass index and waist circumference [114]. *Lactobacillus rhamnosus bv-77*, *Lactobacillus salivarius*, and *Bifidobacterium animalis* were assessed on a pediatric population from China and they were proven not only to significantly reduce body mass index in obese children, but also to cause a significant improvement in blood lipid content [73]. *Bifidobacterium pseudocatenulatum CECT 7765* was recently assessed in a pediatric population with obesity and insulin resistance, and the authors concluded that this probiotic was associated with not only lower levels of C-reactive protein and monocyte chemoattractant protein-1, but also elevated levels of high-density lipoprotein cholesterol and omentin-1, highlighting the major anti-inflammatory effect of this bacteria in the studied population [115]. Contrariwise, a study which assessed the role of synbiotics (*Bifidobacterium bifidum*, *Bifidobacterium longum*, *Lactobacillus acidophilus*, *Lacticaseibacillus rhamnosus*, *Enterococcus faecium*, and fructo-oligosaccharides) failed to prove any effect on lipid parameters and glucose metabolism [89] (Table 2).

**Table 2:** Implications of prebiotics, probiotics, and symbiotic in obesity-related complications.

Reference (Author, Year)	Type of Study	Study Population	Objectives	Prebiotics, Probiotics and Synbiotic	Obesity			Conclusions
					NAFLD/ NASH	Metabolic Syndrome	Insulin Resistance	
Loomba et al., 2017 [97]	Prospective study	*n* = 86 NAFLD Age: >18 years	Identification of fecal-microbiome-derived metagenomic signature to detect advanced fibrosis in NAFLD	−	+	−	−	*Escherichia*, *Enterobacteria*, *Proteobacteria*, and *Bacteroides* are more commonly found in patients with NASH.
Vajro et al., 2011 [70]	Double-blind, placebo-controlled pilot study	*n* = 20 (10 probiotic/10 placebo) Age: 9–13 years BMI > 95th percentile	Effects of short-term probiotic on children with obesity-related liver disease	*Lactobacillus rhamnosus GG* (12 billion CFU/day) for 8 weeks	+	−	−	*Lactobacillus rhamnosus* decrease of ALT in children with hepatic steatosis.
Alisi et al., 2014 [98]	Parallel-arm double-blind trial	*n* = 44 (22 VSL#3/22 placebo) Age: 9–13 years BMI > 85th percentile Obese + NAFLD	Effect of *VSL#3* on structural improvement of FL in obese children with biopsy-proven NAFLD	*VSL#3* is a mixture of *Streptococcus thermophilus*, *Bifidobacteria breve*, *Bifidobacteria infantis*, *Bifidobacteria longum*, *Lactobacillus acidophilus*, *L. plantarum*, *L. paracasei*, and *L. delbrueckii* subsp. *bulgaricus* 1 sachet/day of VSL#3 < 10 years, 2 sachets/day of VSL#3 > 10 years vs. placebo for 4 months	+	−	−	*Streptococcus thermophilus*, *lactobacilli*, and *bifidobacteria* might improve the ultrasound of the liver after 4 months of administration.
Famouri et al., 2017 [99]	Randomized, triple-blind, placebo-controlled trial	*n* = 64 (32 probiotic/32 placebo) Age: 10–18 years BMI > 85th percentile Obese + NAFLD	Effects of probiotics on NAFLD in obese children and adolescents	*Lactobacillus acidophilus ATCC B3208*, 3 × 10^9^ CFU; *Bifidobacterium lactis DSMZ 32269*, 6 × 10^9^ CFU; *Bifidobacterium bifidum ATCC SD6576*, 2 × 10^9^ CFU; *Lactobacillus rhamnosus DSMZ 21690*, 2 × 10^9^ CFU 1 caps/day vs. placebo for 12 weeks	+	−	−	*Lactobacillus rhamnosus*, *Lactobacillus acidophilus*, *Bifidobacterium lactis*, and *Bifidobacterium bifidum* for 12 weeks in obese children → improving AST, ALT level, liver steatosis, and lipid profile.
Rodrigo et al., 2022 [100]	Double-blind, randomized, placebo-controlled trial	*n* = 84 (43 probiotic/41 placebo) Age: 5–15 years BMI > +2 standard deviation for age AST/ALT < 1 Hepatic steatosis, grade I to III	Effects of probiotics on metabolic derangement in obese children with nonalcoholic fatty liver disease/nonalcoholic steatohepatitis (NAFLD/NASH).	BioKult 14 strain probiotic capsule: 1 caps/day < 12 years, 2 caps/day ≥ 12 years 6 months	+	−	−	Significant decrease of body mass index irrespective of the group which received probiotic with 14 bacterial strains or the placebo group.
Kilic Yildirim et al., 2022 [89]	Randomized, double-blind, placebo-controlled trial	*n* = 61 (30 synbiotic/31 placebo) Age: 8–17 years BMI > 95th percentile	Effects of synbiotic on anthropometric measurements, glucose metabolism, and lipid parameters	Synbiotic including *Lacticaseibacillus rhamnosus*, *Lactobacillus acidophilus*, *Bifidobacterium longum*, *Bifidobacterium bifidum*, and *Enterococcus faecium*, combined with fructo-oligosaccharides for 12 weeks vs. placebo.	No impact on NAFLD	−	−	Synbiotics (*Bifidobacterium longum*, *Bifidobacterium bifidum*, *Enterococcus faecium*, *Lactobacillus acidophilus*, *Lacticaseibacillus rhamnosus*, and fructo-oligosaccharides) → no impact on NAFLD and no effect on lipid parameters and glucose metabolism.
Karlsson et al., 2015 [75,77]	Double-blind, randomized, placebo-controlled intervention trial	*n* = 120 (58 probiotic/62 placebo) Age: 8–9 years	Long-term effect of feeding with *Lactobacillus paracasei F19 (LF19)* on body composition, growth, and metabolic markers (TC, HDLC, apo A-1, apo B, TG, glucose, AST, ALT, S-insuline)	Administration of *Lactobacillus paracasei F19* from 4 to 13 months of age, 1 × 10^8^ CFU/at least once daily	−	No effect	−	*Lactobacillus rhamnosus GG* and *Lactobacillus paracasei* ssp. *F19* → no effect on metabolic syndrome.
Benítez-Páez et al., 2020 [110]	Cross-sectional study	*n*= 51 (16 lean/20 obese/15 obese + IR Age: 5–17 years BMI z-score of ≥2 SD	Study of gut microbiota profile of lean and obese children with/without insulin resistance and associations with specific obesity-related complications and metabolic inflammation	−	−	+	−	*Blautia species* → modulating glucose metabolism in children. Depletion of *B. luti* and *B. wexlerae* species occur in obesity and lead to metabolic inflammation and insulin resistance.
Solito et al., 2023 [114]	Cross-over, double-blind, randomized control trial	*n*= 101 (51 probiotic/50 placebo) obese+ IR Age: 6–18 years	Impact of a probiotic supplementation in pediatric obesity on weight, metabolic alterations, and selected gut microbial groups	2 × 10^9^ CFU/AFU/day of *Bifidobacterium breve BR03 (DSM 16604)* and *B. breve B632 (DSM 24706)* or placebo for 8 weeks	−	Positive effect	Improve insulin sensitivity	*Bifidobacterium breve B632* and *Bifidobacterium breve BR03* administered for 8 weeks → improvement in insulin sensitivity and decreased body mass index and waist circumference.
Chen et al., 2022 [73]	Double-blind, randomized, placebo-controlled trial	*n* = 53 (27 probiotic/26 placebo)	Effects of multi-strain probiotics on the gut microbiota and weight control/BMI, LDLC, HDLC, adiponectin, and leptin	Multi-strain probiotics consisting of *Lactobacillus salivarius AP-32*, *Lactobacillus rhamnosus bv-77*, and *Bifidobacterium animalis CP-9* for 12 weeks	−	Positive effect	−	*Lactobacillus salivarius* and *Bifidobacterium animalis* on a pediatric population from China → significantly reduce body mass index in obese children and a significant improvement of blood lipid content
Sanchis-Chordà et al., 2019 [115]	Prospective analytical intervention study	*n* = 56 (28 probiotic/28 control Age: 7–16 years obese + IR BMI z-score of ≥2 SD	Effect of *Bifidobacterium pseudocatenulatum CECT 7765* on inflammatory cytokines, cardiometabolic risk factors, and gut microbiota composition in obese children with IR	*B. pseudocatenulatum (CECT 7765)* 1 × 10^9^–1 × 10^10^ CFU per day for 91 days vs. control	−	+	+	*Bifidobacterium pseudocatenulatum CECT 7765* → determine lower levels of C-reactive protein and monocyte chemoattractant protein-1 and elevated levels of high-density lipoprotein cholesterol and omentin-1 → anti-inflammatory effect

Legend: BMI: body mass index; CFU: colony forming units; FL: fatty liver; HDLC: high-density lipoprotein cholesterol; IR: insulin resistance; LDLC: low-density lipoprotein cholesterol.

## 4. Conclusions

The gut microbiome is not only a current research hotspot in all medical areas, but it is also a core puzzle piece for a complex puzzle that is far from being completely understood, in which obesity and its associated complications represent only a minor part. Taking into account the fact that an early inflammatory status associated with pediatric obesity is no longer a myth and that most of the complications related to this nutritional disorder might occur even during childhood and adolescence, preventive and therapeutic strategies aiming to decrease this inflammation should take priority in the current research world. In spite of the limited and contradictory evidence regarding the role of pre-, pro-, and synbiotics in children with obesity and its associated complications, this topic is definitely worthy of investigation, since it is clearly a major opportunity for developing low-cost effective strategies in the management of childhood obesity, which is a worldwide public health problem.

## Data Availability

Not applicable.
